# Supplementary material for: Protective Efficacy of a Novel DNA Vaccine with a CL264 Molecular Adjuvant against Toxoplasma gondii in a Murine Model
Source: Vaccines (Basel). 2024 May 25;12(6):577. doi: 10.3390/vaccines12060577 (PMC11209281; doi:10.3390/vaccines12060577)
Supplement: Supplementary file 1 [file vaccines-12-00577-s001.zip › vaccines-3000423-supplementary.pdf]

Table S1 Sequences of oligonucleotide primers for PCR

Gene	Primer sequence	Site for endonuclease	Size of DNA fragment
IST	Forward 5'-ATGGATTACAAGGATGACGACG ATAAGCGTTGGTGTGCCCCTGGT-3'<br>Reverse 5'-GACCCGCCGTTCACTACTCG-3'		2673bp
Plasmid backbone	Forward 5'-TAGTGAACGGCGGGTCCCGGAC TCAGATCTCGAGCTC-3'<br>Reverse 5'-GTCATCCTTGTAATCCATCAGCT CTGCTTATATAGACCTCCCA-3'	*BamH* I<br>*Nde* I	4700bp

Dataset S1 DNA sequencing result of recombinant pEGFP-TgIST

```
pEGFP-N1-IST.txt from 1 to 7396
Alignment to
0001_31622031502671_(TgIST-1)_[CMV-F].seq--  Matches:1146; Mismatches:0; Gaps:552; Unattempted:5699

             *         *         *         *         *         *         *         *         *         *
     1>TAGTTATTAATAGTAATCAATTACGGGGTCATTAGTTCATAGCCCATATATGGAGTTCCGCGTTACATAACTTACGGTAAATGGCCCGCCTGGCTGACCG>100
     1>~~~~~~~~~~~~~~~~~~~~~~~CGGGGT-----G------G---------GGAG--C------A-AT--------------------------------->16

             *         *         *         *         *         *         *         *         *         *
   101>CCCAACGACCCCCGCCCATTGACGTCAATAATGACGTATGTTCCCATAGTAACGCCAATAGGGACTTTCCATTGACGTCAATGGGTGGAGTATTTACGGT>200
    16>---------------------------------------------------------------------------------------------------->16

             *         *         *         *         *         *         *         *         *         *
   201>AAACTGCCCACTTGGCAGTACATCAAGTGTATCATATGCCAAGTACGCCCCCTATTGACGTCAATGACGGTAAATGGCCCGCCTGGCATTATGCCCAGTA>300
    16>---------------------------------------------------------------------------------------------------->16

             *         *         *         *         *         *         *         *         *         *
   301>CATGACCTTATGGGACTTTCCTACTTGGCAGTACATCTACGTATTAGTCATCGCTATTACCATGGTGATGCGGTTTTGGCAGTACATCAATGGGCGTGGA>400
    16>---------------------------------------------------------------------------------------------------->16

             *         *         *         *         *         *         *         *         *         *
   401>TAGCGGTTTGACTCACGGGGATTTCCAAGTCTCCACCCCATTGACGTCAATGGGAGTTTGTTTTGGCACCAAAATCAACGGGACTTTCCAAAATGTCGTA>500
    16>---------------------------------------------------------------------------------------------------->16

             *         *         *         *         *         *         *         *         *         *
   501>ACAACTCCGCCCCATTGACGCAAATGGGCGGTAGGCGTGTACGGTGGGAGGTCTATATAAGCAGAGCTGatgGATTACAAGGATGACGACGATAAGCGTT>600
    17>-----------------------------------------------------------AGCAGAGCTGATGGATTACAAGGATGACGACGATAAGCGTT>57

             *         *         *         *         *         *         *         *         *         *
   601>GGTGTGCCCCTGGTGAAACGATAGAGAAGCAAAACCAAATGGAAGTCGGTGAGCCTCCGCAGATAAGTGGGCGCGAAGCGGGAGTTCTCGCAGGAGCTCC>700
    58>GGTGTGCCCCTGGTGAAACGATAGAGAAGCAAAACCAAATGGAAGTCGGTGAGCCTCCGCAGATAAGTGGGCGCGAAGCGGGAGTTCTCGCAGGAGCTCC>157

             *         *         *         *         *         *         *         *         *         *
   701>TCTTGGCGCTCGCGATGGATTCCGTAGTAGTACAAGCAGTTGTTGTCACGCACTTCGATTTAGAAGATCTGGACCGTATGTGTTTAGTTGTCTTGCTGTG>800
   158>TCTTGGCGCTCGCGATGGATTCCGTAGTAGTACAAGCAGTTGTTGTCACGCACTTCGATTTAGAAGATCTGGACCGTATGTGTTTAGTTGTCTTGCTGTG>257

             *         *         *         *         *         *         *         *         *         *
   801>GGATTAATTGCCATTTCATGCGCACCAGAGAGTTTGTGTTTTGAGGCAAGTCTTCCCTTCCTGTTGGCTGGACTTGCGGGTTCAGAGGCTATCGCAGATG>900
   258>GGATTAATTGCCATTTCATGCGCACCAGAGAGTTTGTGTTTTGAGGCAAGTCTTCCCTTCCTGTTGGCTGGACTTGCGGGTTCAGAGGCTATCGCAGATG>357
```

```
              *         *         *         *         *         *         *         *         *         *
 901>GCGGCCATCTAGCCGTATATGCAGGGACTGTGACGGCCTCTGCCCGCGGTGGAACTCACTGGTCCCAGAGAGAGGCTCTTGTCACGGCCCCGATGCAGGC>1000
 358>GCGGCCATCTAGCCGTATATGCAGGGACTGTGACGGCCTCTGCCCGCGGTGGAACTCACTGGTCCCAGAGAGAGGCTCTTGTCACGGCCCCGATGCAGGC>457

              *         *         *         *         *         *         *         *         *         *
1001>CAGGCGCTTGGCGGCAGAGGGTGGAAGTGAATCAGAAGACGAGCAGGGTGTCGCGAGGCCGGGAGGAACCACGGCGACACCCAGCACGCCACTGCCACCG>1100
 458>CAGGCGCTTGGCGGCAGAGGGTGGAAGTGAATCAGAAGACGAGCAGGGTGTCGCGAGGCCGGGAGGAACCACGGCGACACCCAGCACGCCACTGCCACCG>557

              *         *         *         *         *         *         *         *         *         *
1101>CGGCCGTCTGTGCTGATGGAGCTACGCTCTCCTTCCACTCCAGGTGGTTCCGAACCAGCCTCGTCTGCGGCCCTGCAGGCGGGGTCTTTGTCTGCTCCCC>1200
 558>CGGCCGTCTGTGCTGATGGAGCTACGCTCTCCTTCCACTCCAGGTGGTTCCGAACCAGCCTCGTCTGCGGCCCTGCAGGCGGGGTCTTTGTCTGCTCCCC>657

              *         *         *         *         *         *         *         *         *         *
1201>AATTAATTCAGTCACAGCGGAGCCCGACAGGATCGTCTTCACCTTTACTTAGGACATCCATGCAGGAGTCCTCTGAACAGCCTGGATCACCCTCCACAAA>1300
 658>AATTAATTCAGTCACAGCGGAGCCCGACAGGATCGTCTTCACCTTTACTTAGGACATCCATGCAGGAGTCCTCTGAACAGCCTGGATCACCCTCCACAAA>757

              *         *         *         *         *         *         *         *         *         *
1301>GCCCGGTAGCCCATCCGGACACGCGAACAAGAGTCCGCCTCTACCCCTACAGGAACAAGGTCGCGCTGTACCTTCTGGGGACCAACCTCCTCCGCAGCCG>1400
 758>GCCCGGTAGCCCATCCGGACACGCGAACAAGAGTCCGCCTCTACCCCTACAGGAACAAGGTCGCGCTGTACCTTCTGGG-ACCAACCTCCTCCGCAGCCG>856

              *         *         *         *         *         *         *         *         *         *
1401>CCTCCACCTCATTCACCGCCACGTGTGGCTACGGGCAGAGAAATCCGAGAACGGGTGCTCAGAGAGCTAGAACTGAGACGGGGTTCAGGTGGGGAAACGT>1500
 857>CCTCCACCTCATTCACCGCCACGTGTGGCTACGGGCAGAGAAATCCGAGAACGGGTGCTCAGAGAGCTAGAACTGAGACGGGGTTCAGGTGGGGAAACGT>956

              *         *         *         *         *         *         *         *         *         *
1501>CATCAAGGCTCAGACCTCCTGCCAAACGGCCAAGCGAGGAGGCTCGCGGCTTTGACAGACCGGGGACTACAGGAACTGAAGGACCGCGTGTTCGGACCGC>1600
 957>CATCAAGGCTCAGACCTCCTGCCAAACGGCCAAGCGAGGAGGCTCGCGGCTTTGACAGACCGGGGACTACAGGAACTGAAGGACCGCGTGTTCG-A-CGC>1054

              *         *         *         *          *         *         *         *         *
1601>GCTAGATGTTTTCCGCGAGATGACTCAACAAACAAAACGACTAAAGCTC-ACGGCAGAGTCGTCCTCCAGCGCGACAGCAAGCCCAGGTATTGACAGACC>1699
1055>GCTAGATGTTTT-CGCGAGATGACTCAACAAACAAAACGACTAAAGCTCCAC-GCAGAGTCGT-CT-CAGCGCGACAGC-AGCCCAGGTATTGACAGA-->1147

     *         *         *         *         *         *         *         *         *         *
1700>GGGGACTACAGGAACTAAAGGACCGCAAGTTCGGACCGCGCTAGATGTTTTGCGCGAGTTGACTCAACAAGCAAAACAACTAAGGCTCACGGCAGAGTCG>1799
1147>---------------------------------------------------------------------------------------------------->1147

     *         *         *         *         *         *         *         *         *         *
1800>TCCTCCACCGCGACAGCAAGCCCAGCTCCTGAGACCGAACGTCGATCAAGCTCCCCTCGACCGACGCCCCCTCCTCTCCCACGACCAATGTCGCCTATCC>1899
1147>---------------------------------------------------------------------------------------------------->1147

     *         *         *         *         *         *         *         *         *         *
1900>GGACAGGCAAAGAAATCATGGAGAAGGTGAAAAGAGAGCGAGCCCTCCAACGTGCACAAATGGAAAGACGACATCGCACAGAAGGCGACGCAACCTCATC>1999
1147>---------------------------------------------------------------------------------------------------->1147

     *         *         *         *         *         *         *         *         *         *
2000>GGTACAAGGACAAGCGCCTCTTATAGGTCCACGACCACGGCCTAAAAGTGCGCTACAGACTTTTCGTGAGGTGTACGCAGAGATGCGGGCGCGACAAGCC>2099
1147>---------------------------------------------------------------------------------------------------->1147

     *         *         *         *         *         *         *         *         *         *
2100>GCACGACAGGTGCTTTCCAGGCACACATCAACTCCAGCATCTCAGATGCATTCACAACCATATACGTCTTCTCATCCCAGCAAGACGTCGTCACCGAGTC>2199
1147>---------------------------------------------------------------------------------------------------->1147

     *         *         *         *         *         *         *         *         *         *
2200>GTGCGAGTGCAGGTTCTTTGAGAGGCAGTCAACTTCTTCAAGAAGGGCATGGTGGGAAAGGCGCGTCAACGGCTTCTCTACACTCCGAGCCTGGCGCTGC>2299
1147>---------------------------------------------------------------------------------------------------->1147

     *         *         *         *         *         *         *         *         *         *
2300>GTCTATGCCTGGACCTTCGCCTAAATCCGAACCTCCCCTTCTACCAACACAACGCACTGAATGGCCAAGAGAACGGTTCCCCAGCTCGTCCGACCGCGGA>2399
1147>---------------------------------------------------------------------------------------------------->1147

     *         *         *         *         *         *         *         *         *         *
2400>CGTCGTTCTCCTGAAAGGGGTACCCGTGGACCCCTGGGAGAGCCGTCGCATGGCATCGCCAAGATGCTGTATGCAGGCGCCCCGACAACAGTTCAGTCTC>2499
1147>---------------------------------------------------------------------------------------------------->1147

     *         *         *         *         *         *         *         *         *         *
2500>ATCCCGGAAGTGCGTCGCTTGAGGTACCTGCGGCATTCGGAAAAGTTGAAGAGGGTCCTCGTAGGGAACCTCAGCCTTTCGCTGTAATGTTTCCTTTTGC>2599
1147>---------------------------------------------------------------------------------------------------->1147

     *         *         *         *         *         *         *         *         *         *
2600>CACCGTCGCACATAGGGCGGCTATAGGTGGAGAAACTCCGGCAGGCATGTCAGGCCCCGATGGAAACGTAGTTCGACATATTGCTTTGCAGAGAGTGTCT>2699
1147>---------------------------------------------------------------------------------------------------->1147
```

```
          *         *         *         *         *         *         *         *         *         *
2700>TTGTCTCTGCCCCCGCGGTCCACAGGCACAGCTCCTGAAACGCCAATGCCTCGGGAACGTCGGCCACGCAAACAAGCTAAGCCCACCAGGTATTCAGCTT>2799
1147>---------------------------------------------------------------------------------------------------->1147

          *         *         *         *         *         *         *         *         *         *
2800>CCTATGAACCACACCATCCTAGTTCTCCAGCTCCCACACCCACTGTTTCTGGGCCGCTCATTTCGGCGTCGACTGGTCACTCGCCCCCGCAGACGAGTTC>2899
1147>---------------------------------------------------------------------------------------------------->1147

          *         *         *         *         *         *         *         *         *         *
2900>CTCTGCCTCGCCTCCGTTCCCCTCCCAGAGCAGCTGGCAACCAACAGGTCAACCTCTCACGCAAAGACTCACCTTACCTGGACAGAGCGTCTTGAGGCAG>2999
1147>---------------------------------------------------------------------------------------------------->1147

          *         *         *         *         *         *         *         *         *         *
3000>ATGGCTCCAGTTCCCCCTGCGCCTCGAGGAGGCGGCCCTGCTTTTCCGCCACAAGAGGAACCCTTGAACTTGTGTACACATGCGAGGAATCTGCCTCGTC>3099
1147>---------------------------------------------------------------------------------------------------->1147

          *         *         *         *         *         *         *         *         *         *
3100>TTCCCCCGGCATCGGTGATCGCCCATCAATTGGGTGCCAGTGCAGCTGTTTCAGCTCCGGGTGTGAGTCCCAGTGTTGGGTCAGCCCCTTCGGCGTCTCC>3199
1147>---------------------------------------------------------------------------------------------------->1147

          *         *         *         *         *         *         *         *         *         *
3200>ACCTGTGCGATTGCCGTCACCGCCATCGCTTCATCGCCGGCCGCCGAGTAGTGAACGGCGGGTCCCGGACTCAGATCTCGAGCTCAAGCTTCGAATTCTG>3299
1147>---------------------------------------------------------------------------------------------------->1147

          *         *         *         *         *         *         *         *         *         *
3300>CAGTCGACGGTACCGCGGGCCCGGGATCCACCGGTCGCCACCATGGTGAGCAAGGGCGAGGAGCTGTTCACCGGGGTGGTGCCCATCCTGGTCGAGCTGG>3399
1147>---------------------------------------------------------------------------------------------------->1147

          *         *         *         *         *         *         *         *         *         *
3400>ACGGCGACGTAAACGGCCACAAGTTCAGCGTGTCCGGCGAGGGCGAGGGCGATGCCACCTACGGCAAGCTGACCCTGAAGTTCATCTGCACCACCGGCAA>3499
1147>---------------------------------------------------------------------------------------------------->1147
```

```
          *         *         *         *         *         *         *         *         *         *
3500>GCTGCCCGTGCCCTGGCCCACCCTCGTGACCACCCTGACCTACGGCGTGCAGTGCTTCAGCCGCTACCCCGACCACATGAAGCAGCACGACTTCTTCAAG>3599
1147>---------------------------------------------------------------------------------------------------->1147

          *         *         *         *         *         *         *         *         *         *
3600>TCCGCCATGCCCGAAGGCTACGTCCAGGAGCGCACCATCTTCTTCAAGGACGACGGCAACTACAAGACCCGCGCCGAGGTGAAGTTCGAGGGCGACACCC>3699
1147>---------------------------------------------------------------------------------------------------->1147

          *         *         *         *         *         *         *         *         *         *
3700>TGGTGAACCGCATCGAGCTGAAGGGCATCGACTTCAAGGAGGACGGCAACATCCTGGGGCACAAGCTGGAGTACAACTACAACAGCCACAACGTCTATAT>3799
1147>---------------------------------------------------------------------------------------------------->1147

          *         *         *         *         *         *         *         *         *         *
3800>CATGGCCGACAAGCAGAAGAACGGCATCAAGGTGAACTTCAAGATCCGCCACAACATCGAGGACGGCAGCGTGCAGCTCGCCGACCACTACCAGCAGAAC>3899
1147>---------------------------------------------------------------------------------------------------->1147

          *         *         *         *         *         *         *         *         *         *
3900>ACCCCCATCGGCGACGGCCCCGTGCTGCTGCCCGACAACCACTACCTGAGCACCCAGTCCGCCCTGAGCAAAGACCCCAACGAGAAGCGCGATCACATGG>3999
1147>---------------------------------------------------------------------------------------------------->1147

          *         *         *         *         *         *         *         *         *         *
4000>TCCTGCTGGAGTTCGTGACCGCCGCCGGGATCACTCTCGGCATGGACGAGCTGTACAAGTAAAGCGGCCGCGACTCTAGATCATAATCAGCCATACCACA>4099
1147>---------------------------------------------------------------------------------------------------->1147

          *         *         *         *         *         *         *         *         *         *
4100>TTTGTAGAGGTTTTACTTGCTTTAAAAAACCTCCCACACCTCCCCCTGAACCTGAAACATAAAATGAATGCAATTGTTGTTGTTAACTTGTTTATTGCAG>4199
1147>---------------------------------------------------------------------------------------------------->1147

          *         *         *         *         *         *         *         *         *         *
4200>CTTATAATGGTTACAAATAAAGCAATAGCATCACAAATTTCACAAATAAAGCATTTTTTTCACTGCATTCTAGTTGTGGTTTGTCCAAACTCATCAATGT>4299
1147>---------------------------------------------------------------------------------------------------->1147

          *         *         *         *         *         *         *         *         *         *
4300>ATCTTAAGGCGTAAATTGTAAGCGTTAATATTTTGTTAAAATTCGCGTTAAATTTTTGTTAAATCAGCTCATTTTTTAACCAATAGGCCGAAATCGGCAA>4399
1147>---------------------------------------------------------------------------------------------------->1147

          *         *         *         *         *         *         *         *         *         *
4400>AATCCCTTATAAATCAAAAGAATAGACCGAGATAGGGTTGAGTGTTGTTCCAGTTTGGAACAAGAGTCCACTATTAAAGAACGTGGACTCCAACGTCAAA>4499
1147>---------------------------------------------------------------------------------------------------->1147
```

```
     *         *         *         *         *         *         *         *         *         *
4500>GGGCGAAAAACCGTCTATCAGGGCGATGGCCCACTACGTGAACCATCACCCTAATCAAGTTTTTTGGGGTCGAGGTGCCGTAAAGCACTAAATCGGAACC>4599
1147>---------------------------------------------------------------------------------------------------->1147

     *         *         *         *         *         *         *         *         *         *
4600>CTAAAGGGAGCCCCCGATTTAGAGCTTGACGGGGAAAGCCGGCGAACGTGGCGAGAAAGGAAGGGAAGAAAGCGAAAGGAGCGGGCGCTAGGGCGCTGGC>4699
1147>---------------------------------------------------------------------------------------------------->1147

     *         *         *         *         *         *         *         *         *         *
4700>AAGTGTAGCGGTCACGCTGCGCGTAACCACCACACCCGCCGCGCTTAATGCGCCGCTACAGGGCGCGTCAGGTGGCACTTTTCGGGGAAATGTGCGCGGA>4799
1147>---------------------------------------------------------------------------------------------------->1147

     *         *         *         *         *         *         *         *         *         *
4800>ACCCCTATTTGTTTATTTTTCTAAATACATTCAAATATGTATCCGCTCATGAGACAATAACCCTGATAAATGCTTCAATAATATTGAAAAAGGAAGAGTC>4899
1147>---------------------------------------------------------------------------------------------------->1147

     *         *         *         *         *         *         *         *         *         *
4900>CTGAGGCGGAAAGAACCAGCTGTGGAATGTGTGTCAGTTAGGGTGTGGAAAGTCCCCAGGCTCCCCAGCAGGCAGAAGTATGCAAAGCATGCATCTCAAT>4999
1147>---------------------------------------------------------------------------------------------------->1147

     *         *         *         *         *         *         *         *         *         *
5000>TAGTCAGCAACCAGGTGTGGAAAGTCCCCAGGCTCCCCAGCAGGCAGAAGTATGCAAAGCATGCATCTCAATTAGTCAGCAACCATAGTCCCGCCCCTAA>5099
1147>---------------------------------------------------------------------------------------------------->1147

     *         *         *         *         *         *         *         *         *         *
5100>CTCCGCCCATCCCGCCCCTAACTCCGCCCAGTTCCGCCCATTCTCCGCCCCATGGCTGACTAATTTTTTTTATTTATGCAGAGGCCGAGGCCGCCTCGGC>5199
1147>---------------------------------------------------------------------------------------------------->1147

     *         *         *         *         *         *         *         *         *         *
5200>CTCTGAGCTATTCCAGAAGTAGTGAGGAGGCTTTTTTGGAGGCCTAGGCTTTTGCAAAGATCGATCAAGAGACAGGATGAGGATCGTTTCGCATGATTGA>5299
1147>---------------------------------------------------------------------------------------------------->1147
```

```
     *         *         *         *         *         *         *         *         *         *
5300>ACAAGATGGATTGCACGCAGGTTCTCCGGCCGCTTGGGTGGAGAGGCTATTCGGCTATGACTGGGCACAACAGACAATCGGCTGCTCTGATGCCGCCGTG>5399
1147>---------------------------------------------------------------------------------------------------->1147

     *         *         *         *         *         *         *         *         *         *
5400>TTCCGGCTGTCAGCGCAGGGGCGCCCGGTTCTTTTTGTCAAGACCGACCTGTCCGGTGCCCTGAATGAACTGCAAGACGAGGCAGCGCGGCTATCGTGGC>5499
1147>---------------------------------------------------------------------------------------------------->1147

     *         *         *         *         *         *         *         *         *         *
5500>TGGCCACGACGGGCGTTCCTTGCGCAGCTGTGCTCGACGTTGTCACTGAAGCGGGAAGGGACTGGCTGCTATTGGGCGAAGTGCCGGGGCAGGATCTCCT>5599
1147>---------------------------------------------------------------------------------------------------->1147

     *         *         *         *         *         *         *         *         *         *
5600>GTCATCTCACCTTGCTCCTGCCGAGAAAGTATCCATCATGGCTGATGCAATGCGGCGGCTGCATACGCTTGATCCGGCTACCTGCCCATTCGACCACCAA>5699
1147>---------------------------------------------------------------------------------------------------->1147

     *         *         *         *         *         *         *         *         *         *
5700>GCGAAACATCGCATCGAGCGAGCACGTACTCGGATGGAAGCCGGTCTTGTCGATCAGGATGATCTGGACGAAGAGCATCAGGGGCTCGCGCCAGCCGAAC>5799
1147>---------------------------------------------------------------------------------------------------->1147

     *         *         *         *         *         *         *         *         *         *
5800>TGTTCGCCAGGCTCAAGGCGAGCATGCCCGACGGCGAGGATCTCGTCGTGACCCATGGCGATGCCTGCTTGCCGAATATCATGGTGGAAAATGGCCGCTT>5899
1147>---------------------------------------------------------------------------------------------------->1147

     *         *         *         *         *         *         *         *         *         *
5900>TTCTGGATTCATCGACTGTGGCCGGCTGGGTGTGGCGGACCGCTATCAGGACATAGCGTTGGCTACCCGTGATATTGCTGAAGAGCTTGGCGGCGAATGG>5999
1147>---------------------------------------------------------------------------------------------------->1147

     *         *         *         *         *         *         *         *         *         *
6000>GCTGACCGCTTCCTCGTGCTTTACGGTATCGCCGCTCCCGATTCGCAGCGCATCGCCTTCTATCGCCTTCTTGACGAGTTCTTCTGAGCGGGACTCTGGG>6099
1147>---------------------------------------------------------------------------------------------------->1147

     *         *         *         *         *         *         *         *         *         *
6100>GTTCGAAATGACCGACCAAGCGACGCCCAACCTGCCATCACGAGATTTCGATTCCACCGCCGCCTTCTATGAAAGGTTGGGCTTCGGAATCGTTTTCCGG>6199
1147>---------------------------------------------------------------------------------------------------->1147

     *         *         *         *         *         *         *         *         *         *
6200>GACGCCGGCTGGATGATCCTCCAGCGCGGGGATCTCATGCTGGAGTTCTTCGCCCACCCTAGGGGGAGGCTAACTGAAACACGGAAGGAGACAATACCGG>6299
1147>---------------------------------------------------------------------------------------------------->1147
```

```
          *         *         *         *         *         *         *         *         *         *
6300>AAGGAACCCGCGCTATGACGGCAATAAAAAGACAGAATAAAACGCACGGTGTTGGGTCGTTTGTTCATAAACGCGGGGTTCGGTCCCAGGGCTGGCACTC>6399
1147>---------------------------------------------------------------------------------------------------->1147

          *         *         *         *         *         *         *         *         *         *
6400>TGTCGATACCCCACCGAGACCCCATTGGGGCCAATACGCCCGCGTTTCTTCCTTTTCCCCACCCCACCCCCCAAGTTCGGGTGAAGGCCCAGGGCTCGCA>6499
1147>---------------------------------------------------------------------------------------------------->1147

          *         *         *         *         *         *         *         *         *         *
6500>GCCAACGTCGGGGCGGCAGGCCCTGCCATAGCCTCAGGTTACTCATATATACTTTAGATTGATTTAAAACTTCATTTTTAATTTAAAAGGATCTAGGTGA>6599
1147>---------------------------------------------------------------------------------------------------->1147

          *         *         *         *         *         *         *         *         *         *
6600>AGATCCTTTTTGATAATCTCATGACCAAAATCCCTTAACGTGAGTTTTCGTTCCACTGAGCGTCAGACCCCGTAGAAAAGATCAAAGGATCTTCTTGAGA>6699
1147>---------------------------------------------------------------------------------------------------->1147

          *         *         *         *         *         *         *         *         *         *
6700>TCCTTTTTTTCTGCGCGTAATCTGCTGCTTGCAAACAAAAAAACCACCGCTACCAGCGGTGGTTTGTTTGCCGGATCAAGAGCTACCAACTCTTTTTCCG>6799
1147>---------------------------------------------------------------------------------------------------->1147

          *         *         *         *         *         *         *         *         *         *
6800>AAGGTAACTGGCTTCAGCAGAGCGCAGATACCAAATACTGTCCTTCTAGTGTAGCCGTAGTTAGGCCACCACTTCAAGAACTCTGTAGCACCGCCTACAT>6899
1147>---------------------------------------------------------------------------------------------------->1147

          *         *         *         *         *         *         *         *         *         *
6900>ACCTCGCTCTGCTAATCCTGTTACCAGTGGCTGCTGCCAGTGGCGATAAGTCGTGTCTTACCGGGTTGGACTCAAGACGATAGTTACCGGATAAGGCGCA>6999
1147>---------------------------------------------------------------------------------------------------->1147

          *         *         *         *         *         *         *         *         *         *
7000>GCGGTCGGGCTGAACGGGGGGTTCGTGCACACAGCCCAGCTTGGAGCGAACGACCTACACCGAACTGAGATACCTACAGCGTGAGCTATGAGAAAGCGCC>7099
1147>---------------------------------------------------------------------------------------------------->1147

          *         *         *         *         *         *         *         *         *         *
7100>ACGCTTCCCGAAGGGAGAAAGGCGGACAGGTATCCGGTAAGCGGCAGGGTCGGAACAGGAGAGCGCACGAGGGAGCTTCCAGGGGGAAACGCCTGGTATC>7199
1147>---------------------------------------------------------------------------------------------------->1147

          *         *         *         *         *         *         *         *         *         *
7200>TTTATAGTCCTGTCGGGTTTCGCCACCTCTGACTTGAGCGTCGATTTTTGTGATGCTCGTCAGGGGGGCGGAGCCTATGGAAAAACGCCAGCAACGCGGC>7299
1147>---------------------------------------------------------------------------------------------------->1147

          *         *         *         *         *         *         *         *         *         *
7300>CTTTTTACGGTTCCTGGCCTTTTGCTGGCCTTTTGCTCACATGTTCTTTCCTGCGTTATCCCCTGATTCTGTGGATAACCGTATTACCGCCATGCAT>7396
1148>-------------------------------------------------------ACGGGACTACAGACTAAAGACCGCAGTTCGACCGCGCCTAGA>1190
```

```
pEGFP-N1-IST.txt from 1 to 7396
Alignment to
0002_31622031502671_(TgIST-1)_[EGFP-N].seq--  Matches:1191; Mismatches:3; Gaps:6203; Unattempted:0

                *         *         *         *         *         *         *         *         *         *
   1>TAGTTATTAATAGTAATCAATTACGGGGTCATTAGTTCATAGCCCATATATGGAGTTCCGCGTTACATAACTTACGGTAAATGGCCCGCCTGGCTGACCG>100
   1>~~~~~~~~~~~~~~~~~C-----C----TC------TCAT--CCCA------------------------------------------------------>12

                *         *         *         *         *         *         *         *         *         *
 101>CCCAACGACCCCCGCCCATTGACGTCAATAATGACGTATGTTCCCATAGTAACGCCAATAGGGACTTTCCATTGACGTCAATGGGTGGAGTATTTACGGT>200
  12>---------------------------------------------------------------------------------------------------->12

                *         *         *         *         *         *         *         *         *         *
 201>AAACTGCCCACTTGGCAGTACATCAAGTGTATCATATGCCAAGTACGCCCCCTATTGACGTCAATGACGGTAAATGGCCCGCCTGGCATTATGCCCAGTA>300
  12>---------------------------------------------------------------------------------------------------->12

                *         *         *         *         *         *         *         *         *         *
 301>CATGACCTTATGGGACTTTCCTACTTGGCAGTACATCTACGTATTAGTCATCGCTATTACCATGGTGATGCGGTTTTGGCAGTACATCAATGGGCGTGGA>400
  12>---------------------------------------------------------------------------------------------------->12

                *         *         *         *         *         *         *         *         *         *
 401>TAGCGGTTTGACTCACGGGGATTTCCAAGTCTCCACCCCATTGACGTCAATGGGAGTTTGTTTTGGCACCAAAATCAACGGGACTTTCCAAAATGTCGTA>500
  12>---------------------------------------------------------------------------------------------------->12

                *         *         *         *         *         *         *         *         *         *
 501>ACAACTCCGCCCCATTGACGCAAATGGGCGGTAGGCGTGTACGGTGGGAGGTCTATATAAGCAGAGCTGatgGATTACAAGGATGACGACGATAAGCGTT>600
  12>---------------------------------------------------------------------------------------------------->12

                *         *         *         *         *         *         *         *         *         *
 601>GGTGTGCCCCTGGTGAAACGATAGAGAAGCAAAACCAAATGGAAGTCGGTGAGCCTCCGCAGATAAGTGGGCGCGAAGCGGGAGTTCTCGCAGGAGCTCC>700
  12>---------------------------------------------------------------------------------------------------->12

                *         *         *         *         *         *         *         *         *         *
 701>TCTTGGCGCTCGCGATGGATTCCGTAGTAGTACAAGCAGTTGTTGTCACGCACTTCGATTTAGAAGATCTGGACCGTATGTGTTTAGTTGTCTTGCTGTG>800
  12>---------------------------------------------------------------------------------------------------->12

                *         *         *         *         *         *         *         *         *         *
 801>GGATTAATTGCCATTTCATGCGCACCAGAGAGTTTGTGTTTTGAGGCAAGTCTTCCCTTCCTGTTGGCTGGACTTGCGGGTTCAGAGGCTATCGCAGATG>900
  12>---------------------------------------------------------------------------------------------------->12

                *         *         *         *         *         *         *         *         *         *
 901>GCGGCCATCTAGCCGTATATGCAGGGACTGTGACGGCCTCTGCCCGCGGTGGAACTCACTGGTCCCAGAGAGAGGCTCTTGTCACGGCCCCGATGCAGGC>1000
  12>---------------------------------------------------------------------------------------------------->12

                *         *         *         *         *         *         *         *         *         *
1001>CAGGCGCTTGGCGGCAGAGGGTGGAAGTGAATCAGAAGACGAGCAGGGTGTCGCGAGGCCGGGAGGAACCACGGCGACACCCAGCACGCCACTGCCACCG>1100
  12>---------------------------------------------------------------------------------------------------->12

                *         *         *         *         *         *         *         *         *         *
1101>CGGCCGTCTGTGCTGATGGAGCTACGCTCTCCTTCCACTCCAGGTGGTTCCGAACCAGCCTCGTCTGCGGCCCTGCAGGCGGGGTCTTTGTCTGCTCCCC>1200
  12>---------------------------------------------------------------------------------------------------->12

                *         *         *         *         *         *         *         *         *         *
1201>AATTAATTCAGTCACAGCGGAGCCCGACAGGATCGTCTTCACCTTTACTTAGGACATCCATGCAGGAGTCCTCTGAACAGCCTGGATCACCCTCCACAAA>1300
  12>---------------------------------------------------------------------------------------------------->12

                *         *         *         *         *         *         *         *         *         *
1301>GCCCGGTAGCCCATCCGGACACGCGAACAAGAGTCCGCCTCTACCCCTACAGGAACAAGGTCGCGCTGTACCTTCTGGGGACCAACCTCCTCCGCAGCCG>1400
  12>---------------------------------------------------------------------------------------------------->12

                *         *         *         *         *         *         *         *         *         *
1401>CCTCCACCTCATTCACCGCCACGTGTGGCTACGGGCAGAGAAATCCGAGAACGGGTGCTCAGAGAGCTAGAACTGAGACGGGGTTCAGGTGGGAAACGT>1500
  12>---------------------------------------------------------------------------------------------------->12

                *         *         *         *         *         *         *         *         *         *
1501>CATCAAGGCTCAGACCTCCTGCCAAACGGCCAAGCGAGGAGGCTCGCGGCTTTGACAGACCGGGGACTACAGGAACTGAAGGACCGCGTGTTCGGACCGC>1600
  12>---------------------------------------------------------------------------------------------------->12

                *         *         *         *         *         *         *         *         *         *
1601>GCTAGATGTTTTCCGCGAGATGACTCAACAAACAAAACGACTAAAGCTCACGGCAGAGTCGTCCTCCAGCGCGACAGCAAGCCCAGGTATTGACAGACCG>1700
  12>---------------------------------------------------------------------------------------------------->12
```

```
                *         *         *         *         *         *         *         *         *         *
1701>GGGACTACAGGAACTAAAGGACCGCAAGTTCGGACCGCGCTAGATGTTTTGCGCGAGTTGACTCAACAAGCAAAACAACTAAGGCTCACGGCAGAGTCGT>1800
  12>---------------------------------------------------------------------------------------------------->12

                *         *         *         *         *         *         *         *         *         *
1801>CCTCCACCGCGACAGCAAGCCCAGCTCCTGAGACCGAACGTCGATCAAGCTCCCCTCGACCGACGCCCCCTCCTCTCCCACGACCAATGTCGCCTATCCG>1900
  12>---------------------------------------------------------------------------------------------------->12

                *         *         *         *         *         *         *         *         *         *
1901>GACAGGCAAAGAAATCATGGAGAAGGTGAAAAGAGAGCGAGCCCTCCAACGTGCACAAATGGAAAGACGACATCGCACAGAAGGCGACGCAACCTCATCG>2000
  12>---------------------------------------------------------------------------------------------------->12

                *         *         *         *         *         *         *         *         *         *
2001>GTACAAGGACAAGCGCCTCTTATAGGTCCACGACCACGGCCTAAAAGTGCGCTACAGACTTTTCGTGAGGTGTACGCAGAGATGCGGGCGCGACAAGCCG>2100
  12>---------------------------------------------------------------------------------------------------->12

                *         *         *         *         *         *         *         *         *         *
2101>CACGACAGGTGCTTTCCAGGCACACATCAACTCCAGCATCTCAGATGCATTCACAACCATATACGTCTTCTCATCCCAGCAAGACGTCGTCACCGAGTCG>2200
  13>-----------------------------------------------------------------------------AGCAAGACGTCGTCACCGAGTCG>35

                *         *         *         *         *         *         *         *         *         *
2201>TGCGAGTGCAGGTTCTTTGAGAGGCAGTCAACTTCTTCAAGAAGGGCATGGTGGGAAAGGCGCGTCAACGGCTTCTCTACACTCCGAGCCTGGCGCTGCG>2300
  36>TGCGAGTGCAG-TTC-TTGAGAGGCAGTC-AC-TC-TCCAGAA-TGCAT-GT-GGAAA-GCGCGTC-ACGGC-TCTCTACACTCCGAG-CT-GCGCTGCG>122

                *         *         *         *         *         *         *         *         *
2301>TCTATGCCTGGACCTTCGCCTAAATCCGAACCTCCCCTTC-TACCAACACAACGCACTGAATGGCCAAGAGAACGGTTCCCAGCTCGTCCGACCGCGGA>2399
 123>TCTATGCCTGGAC-GTCGCCTAAATCCGAACCTCCCCTTCTTACCAACACAACGCACTGAATG-CCAAGAGAACGGTT-CCCAGCTCGTCCGACCGCGGA>219

      *         *         *         *         *         *         *         *         *         *
2400>CGTCGTTCTCCTGAAAGGGGTACCCGTGGACCCCTGGGAGAGCCGTCGCATGGCATCGCCAAGATGCTGTATGCAGGCGCCCCGACAACAGTTCAGTCTC>2499
 220>CGTCGTTCTCCTGAAAGGGGTACCCGTGGACCCCTGGGAGAGCCGTCGCATGGCATCGCCAAGATGCTGTATGCAGGCGCCCCGACAACAGTTCAGTCTC>319

      *         *         *         *         *         *         *         *         *         *
2500>ATCCCGGAAGTGCGTCGCTTGAGGTACCTGCGGCATTCGGAAAAGTTGAAGAGGGTCCTCGTAGGGAACCTCAGCCTTTCGCTGTAATGTTTCCTTTTGC>2599
 320>ATCCCGGAAGTGCGTCGCTTGAGGTACCTGCGGCATTCGGAAAAGTTGAAGAGGGTCCTCGTAGGGAACCTCAGCCTTTCGCTGTAATGTTTCCTTTTGC>419

      *         *         *         *         *         *         *         *         *         *
2600>CACCGTCGCACATAGGGCGGCTATAGGTGGAGAAACTCCGGCAGGCATGTCAGGCCCCGATGGAAACGTAGTTCGACATATTGCTTTGCAGAGAGTGTCT>2699
 420>CACCGTCGCACATAGGGCGGCTATAGGTGGAGAAACTCCGGCAGGCATGTCAGGCCCCGATGGAAACGTAGTTCGACATATTGCTTTGCAGAGAGTGTCT>519
```

```
      *         *         *         *         *         *         *         *         *         *
2700>TTGTCTCTGCCCCCGCGGTCCACAGGCACAGCTCCTGAAACGCCAATGCCTCGGGAACGTCGGCCACGCAAACAAGCTAAGCCCACCAGGTATTCAGCTT>2799
 520>TTGTCTCTGCCCCCGCGGTCCACAGGCACAGCTCCTGAAACGCCAATGCCTCGGGAACGTCGGCCACGCAAACAAGCTAAGCCCACCAGGTATTCAGCTT>619

      *         *         *         *         *         *         *         *         *         *
2800>CCTATGAACCACACCATCCTAGTTCTCCAGCTCCCACACCCACTGTTTCTGGGCCGCTCATTTCGGCGTCGACTGGTCACTCGCCCCCGCAGACGAGTTC>2899
 620>CCTATGAACCACACCATCCTAGTTCTCCAGCTCCCACACCCACTGTTTCTGGGCCGCTCATTTCGGCGTCGACTGGTCACTCGCCCCCGCAGACGAGTTC>719

      *         *         *         *         *         *         *         *         *         *
2900>CTCTGCCTCGCCTCCGTTCCCCTCCCAGAGCAGCTGGCAACCAACAGGTCAACCTCTCACGCAAAGACTCACCTTACCTGGACAGAGCGTCTTGAGGCAG>2999
 720>CTCTGCCTCGCCTCCGTTCCCCTCCCAGAGCAGCTGGCAACCAACAGGTCAACCTCTCACGCAAAGACTCACCTTACCTGGACAGAGCGTCTTGAGGCAG>819

      *         *         *         *         *         *         *         *         *         *
3000>ATGGCTCCAGTTCCCCCTGCGCCTCGAGGAGGCGGCCCTGCTTTTCCGCCACAAGAGGAACCCTTGAACTTGTGTACACATGCGAGGAATCTGCCTCGTC>3099
 820>ATGGCTCCAGTTCCCCCTGCGCCTCGAGGAGGCGGCCCTGCTTTTCCGCCACAAGAGGAACCCTTGAACTTGTGTACACATGCGAGGAATCTGCCTCGTC>919

      *         *         *         *         *         *         *         *         *         *
3100>TTCCCCCGGCATCGGTGATCGCCCATCAATTGGGTGCCAGTGCAGCTGTTTCAGCTCCGGGTGTGAGTCCCAGTGTTGGGTCAGCCCCTTCGGCGTCTCC>3199
 920>TTCCCCCGGCATCGGTGATCGCCCATCAATTGGGTGCCAGTGCAGCTGTTTCAGCTCCGGGTGTGAGTCCCAGTGTTGGGTCAGCCCCTTCGGCGTCTCC>1019

      *         *         *         *         *         *         *         *         *         *
3200>ACCTGTGCGATTGCCGTCACCGCCATCGCTTCATCGCCGGCCGCCGAGTAGTGAACGGCGGGTCCCGGACTCAGATCTCGAGCTCAAGCTTCGAATTCTG>3299
1020>ACCTGTGCGATTGCCGTCACCGCCATCGCTTCATCGCCGGCCGCCGAGTAGTGAACGGCGGGTCCCGGACTCAGATCTCGAGCTCAAGCTTCGAATTCTG>1119

      *         *         *         *         *         *         *         *         *         *
3300>CAGTCGACGGTACCGCGGGCCCGGATCCACCGGTCGCCACCATGGTGAGCAAGGGCGAGGAGCTGTTCACCGGGGTGGTGCCCATCCTGGTCGAGCTGG>3399
1120>CAGTCGACGGTACCGCGGGCCCGGATCCACCGGTCGCCACCATGGTGAGCAAGGGCGAGGAGC-G-TCA------T----CC------GG--G-G--G~>1195

      *         *         *         *         *         *         *         *         *         *
3400>ACGGCGACGTAAACGGCCACAAGTTCAGCGTGTCCGGCGAGGGCGAGGGCGATGCCACCTACGGCAAGCTGACCCTGAAGTTCATCTGCACCACCGGCAA>3499
1195>~~~~~~~~~~~~~~~~~~~~~~~~~~~~~~~~~~~~~~~~~~~~~~~~~~~~~~~~~~~~~~~~~~~~~~~~~~~~~~~~~~~~~~~~~~~~~~~~~~~~>1195
```

```
     *         *         *         *         *         *         *         *         *         *
3500>GCTGCCCGTGCCCTGGCCCACCCTCGTGACCACCCTGACCTACGGCGTGCAGTGCTTCAGCCGCTACCCCGACCACATGAAGCAGCACGACTTCTTCAAG>3599
1195>~~~~~~~~~~~~~~~~~~~~~~~~~~~~~~~~~~~~~~~~~~~~~~~~~~~~~~~~~~~~~~~~~~~~~~~~~~~~~~~~~~~~~~~~~~~~~~~~~~~~>1195

     *         *         *         *         *         *         *         *         *         *
3600>TCCGCCATGCCCGAAGGCTACGTCCAGGAGCGCACCATCTTCTTCAAGGACGACGGCAACTACAAGACCCGCGCCGAGGTGAAGTTCGAGGGCGACACCC>3699
1195>~~~~~~~~~~~~~~~~~~~~~~~~~~~~~~~~~~~~~~~~~~~~~~~~~~~~~~~~~~~~~~~~~~~~~~~~~~~~~~~~~~~~~~~~~~~~~~~~~~~~>1195

     *         *         *         *         *         *         *         *         *         *
3700>TGGTGAACCGCATCGAGCTGAAGGGCATCGACTTCAAGGAGGACGGCAACATCCTGGGGCACAAGCTGGAGTACAACTACAACAGCCACAACGTCTATAT>3799
1195>~~~~~~~~~~~~~~~~~~~~~~~~~~~~~~~~~~~~~~~~~~~~~~~~~~~~~~~~~~~~~~~~~~~~~~~~~~~~~~~~~~~~~~~~~~~~~~~~~~~~>1195

     *         *         *         *         *         *         *         *         *         *
3800>CATGGCCGACAAGCAGAAGAACGGCATCAAGGTGAACTTCAAGATCCGCCACAACATCGAGGACGGCAGCGTGCAGCTCGCCGACCACTACCAGCAGAAC>3899
1195>~~~~~~~~~~~~~~~~~~~~~~~~~~~~~~~~~~~~~~~~~~~~~~~~~~~~~~~~~~~~~~~~~~~~~~~~~~~~~~~~~~~~~~~~~~~~~~~~~~~~>1195

     *         *         *         *         *         *         *         *         *         *
3900>ACCCCCATCGGCGACGGCCCCGTGCTGCTGCCCGACAACCACTACCTGAGCACCCAGTCCGCCCTGAGCAAAGACCCCAACGAGAAGCGCGATCACATGG>3999
1195>~~~~~~~~~~~~~~~~~~~~~~~~~~~~~~~~~~~~~~~~~~~~~~~~~~~~~~~~~~~~~~~~~~~~~~~~~~~~~~~~~~~~~~~~~~~~~~~~~~~~>1195

     *         *         *         *         *         *         *         *         *         *
4000>TCCTGCTGGAGTTCGTGACCGCCGCCGGGATCACTCTCGGCATGGACGAGCTGTACAAGTAAAGCGGCCGCGACTCTAGATCATAATCAGCCATACCACA>4099
1195>~~~~~~~~~~~~~~~~~~~~~~~~~~~~~~~~~~~~~~~~~~~~~~~~~~~~~~~~~~~~~~~~~~~~~~~~~~~~~~~~~~~~~~~~~~~~~~~~~~~~>1195

     *         *         *         *         *         *         *         *         *         *
4100>TTTGTAGAGGTTTTACTTGCTTTAAAAAACCTCCCACACCTCCCCCTGAACCTGAAACATAAAATGAATGCAATTGTTGTTGTTAACTTGTTTATTGCAG>4199
1195>~~~~~~~~~~~~~~~~~~~~~~~~~~~~~~~~~~~~~~~~~~~~~~~~~~~~~~~~~~~~~~~~~~~~~~~~~~~~~~~~~~~~~~~~~~~~~~~~~~~~>1195

     *         *         *         *         *         *         *         *         *         *
4200>CTTATAATGGTTACAAATAAAGCAATAGCATCACAAATTTCACAAATAAAGCATTTTTTTCACTGCATTCTAGTTGTGGTTTGTCCAAACTCATCAATGT>4299
1195>~~~~~~~~~~~~~~~~~~~~~~~~~~~~~~~~~~~~~~~~~~~~~~~~~~~~~~~~~~~~~~~~~~~~~~~~~~~~~~~~~~~~~~~~~~~~~~~~~~~~>1195

     *         *         *         *         *         *         *         *         *         *
4300>ATCTTAAGGCGTAAATTGTAAGCGTTAATATTTTGTTAAAATTCGCGTTAAATTTTTGTTAAATCAGCTCATTTTTTAACCAATAGGCCGAAATCGGCAA>4399
1195>~~~~~~~~~~~~~~~~~~~~~~~~~~~~~~~~~~~~~~~~~~~~~~~~~~~~~~~~~~~~~~~~~~~~~~~~~~~~~~~~~~~~~~~~~~~~~~~~~~~~>1195

     *         *         *         *         *         *         *         *         *         *
4400>AATCCCTTATAAATCAAAAGAATAGACCGAGATAGGGTTGAGTGTTGTTCCAGTTTGGAACAAGAGTCCACTATTAAAGAACGTGGACTCCAACGTCAAA>4499
1195>~~~~~~~~~~~~~~~~~~~~~~~~~~~~~~~~~~~~~~~~~~~~~~~~~~~~~~~~~~~~~~~~~~~~~~~~~~~~~~~~~~~~~~~~~~~~~~~~~~~~>1195
```

```
     *         *         *         *         *         *         *         *         *         *
4500>GGGCGAAAAACCGTCTATCAGGGCGATGGCCCACTACGTGAACCATCACCCTAATCAAGTTTTTTGGGGTCGAGGTGCCGTAAAGCACTAAATCGGAACC>4599
1195>~~~~~~~~~~~~~~~~~~~~~~~~~~~~~~~~~~~~~~~~~~~~~~~~~~~~~~~~~~~~~~~~~~~~~~~~~~~~~~~~~~~~~~~~~~~~~~~~~~~~>1195

     *         *         *         *         *         *         *         *         *         *
4600>CTAAAGGGAGCCCCCGATTTAGAGCTTGACGGGGAAAGCCGGCGAACGTGGCGAGAAAGGAAGGGAAGAAAGCGAAAGGAGCGGGCGCTAGGGCGCTGGC>4699
1195>~~~~~~~~~~~~~~~~~~~~~~~~~~~~~~~~~~~~~~~~~~~~~~~~~~~~~~~~~~~~~~~~~~~~~~~~~~~~~~~~~~~~~~~~~~~~~~~~~~~~>1195

     *         *         *         *         *         *         *         *         *         *
4700>AAGTGTAGCGGTCACGCTGCGCGTAACCACCACACCCGCCGCGCTTAATGCGCCGCTACAGGGCGCGTCAGGTGGCACTTTTCGGGGAAATGTGCGCGGA>4799
1195>~~~~~~~~~~~~~~~~~~~~~~~~~~~~~~~~~~~~~~~~~~~~~~~~~~~~~~~~~~~~~~~~~~~~~~~~~~~~~~~~~~~~~~~~~~~~~~~~~~~~>1195

     *         *         *         *         *         *         *         *         *         *
4800>ACCCCTATTTGTTTATTTTTCTAAATACATTCAAATATGTATCCGCTCATGAGACAATAACCCTGATAAATGCTTCAATAATATTGAAAAAGGAAGAGTC>4899
1195>~~~~~~~~~~~~~~~~~~~~~~~~~~~~~~~~~~~~~~~~~~~~~~~~~~~~~~~~~~~~~~~~~~~~~~~~~~~~~~~~~~~~~~~~~~~~~~~~~~~~>1195

     *         *         *         *         *         *         *         *         *         *
4900>CTGAGGCGGAAAGAACCAGCTGTGGAATGTGTGTCAGTTAGGGTGTGGAAAGTCCCCAGGCTCCCCAGCAGGCAGAAGTATGCAAAGCATGCATCTCAAT>4999
1195>~~~~~~~~~~~~~~~~~~~~~~~~~~~~~~~~~~~~~~~~~~~~~~~~~~~~~~~~~~~~~~~~~~~~~~~~~~~~~~~~~~~~~~~~~~~~~~~~~~~~>1195

     *         *         *         *         *         *         *         *         *         *
5000>TAGTCAGCAACCAGGTGTGGAAAGTCCCCAGGCTCCCCAGCAGGCAGAAGTATGCAAAGCATGCATCTCAATTAGTCAGCAACCATAGTCCCGCCCCTAA>5099
1195>~~~~~~~~~~~~~~~~~~~~~~~~~~~~~~~~~~~~~~~~~~~~~~~~~~~~~~~~~~~~~~~~~~~~~~~~~~~~~~~~~~~~~~~~~~~~~~~~~~~~>1195

     *         *         *         *         *         *         *         *         *         *
5100>CTCCGCCCATCCCGCCCCTAACTCCGCCCAGTTCCGCCCATTCTCCGCCCCATGGCTGACTAATTTTTTTTATTTATGCAGAGGCCGAGGCCGCCTCGGC>5199
1195>~~~~~~~~~~~~~~~~~~~~~~~~~~~~~~~~~~~~~~~~~~~~~~~~~~~~~~~~~~~~~~~~~~~~~~~~~~~~~~~~~~~~~~~~~~~~~~~~~~~~>1195

     *         *         *         *         *         *         *         *         *         *
5200>CTCTGAGCTATTCCAGAAGTAGTGAGGAGGCTTTTTTGGAGGCCTAGGCTTTTGCAAAGATCGATCAAGAGACAGGATGAGGATCGTTTCGCATGATTGA>5299
1195>~~~~~~~~~~~~~~~~~~~~~~~~~~~~~~~~~~~~~~~~~~~~~~~~~~~~~~~~~~~~~~~~~~~~~~~~~~~~~~~~~~~~~~~~~~~~~~~~~~~~>1195
```

```
     *         *         *         *         *         *         *         *         *         *
5300>ACAAGATGGATTGCACGCAGGTTCTCCGGCCGCTTGGGTGGAGAGGCTATTCGGCTATGACTGGGCACAACAGACAATCGGCTGCTCTGATGCCGCCGTG>5399
1195>~~~~~~~~~~~~~~~~~~~~~~~~~~~~~~~~~~~~~~~~~~~~~~~~~~~~~~~~~~~~~~~~~~~~~~~~~~~~~~~~~~~~~~~~~~~~~~~~~~~~>1195

     *         *         *         *         *         *         *         *         *         *
5400>TTCCGGCTGTCAGCGCAGGGGCGCCCGGTTCTTTTTGTCAAGACCGACCTGTCCGGTGCCCTGAATGAACTGCAAGACGAGGCAGCGCGGCTATCGTGGC>5499
1195>~~~~~~~~~~~~~~~~~~~~~~~~~~~~~~~~~~~~~~~~~~~~~~~~~~~~~~~~~~~~~~~~~~~~~~~~~~~~~~~~~~~~~~~~~~~~~~~~~~~~>1195

     *         *         *         *         *         *         *         *         *         *
5500>TGGCCACGACGGGCGTTCCTTGCGCAGCTGTGCTCGACGTTGTCACTGAAGCGGGAAGGGACTGGCTGCTATTGGGCGAAGTGCCGGGGCAGGATCTCCT>5599
1195>~~~~~~~~~~~~~~~~~~~~~~~~~~~~~~~~~~~~~~~~~~~~~~~~~~~~~~~~~~~~~~~~~~~~~~~~~~~~~~~~~~~~~~~~~~~~~~~~~~~~>1195

     *         *         *         *         *         *         *         *         *         *
5600>GTCATCTCACCTTGCTCCTGCCGAGAAAGTATCCATCATGGCTGATGCAATGCGGCGGCTGCATACGCTTGATCCGGCTACCTGCCCATTCGACCACCAA>5699
1195>~~~~~~~~~~~~~~~~~~~~~~~~~~~~~~~~~~~~~~~~~~~~~~~~~~~~~~~~~~~~~~~~~~~~~~~~~~~~~~~~~~~~~~~~~~~~~~~~~~~~>1195

     *         *         *         *         *         *         *         *         *         *
5700>GCGAAACATCGCATCGAGCGAGCACGTACTCGGATGGAAGCCGGTCTTGTCGATCAGGATGATCTGGACGAAGAGCATCAGGGGCTCGCGCCAGCCGAAC>5799
1195>~~~~~~~~~~~~~~~~~~~~~~~~~~~~~~~~~~~~~~~~~~~~~~~~~~~~~~~~~~~~~~~~~~~~~~~~~~~~~~~~~~~~~~~~~~~~~~~~~~~~>1195

     *         *         *         *         *         *         *         *         *         *
5800>TGTTCGCCAGGCTCAAGGCGAGCATGCCCGACGGCGAGGATCTCGTCGTGACCCATGGCGATGCCTGCTTGCCGAATATCATGGTGGAAAATGGCCGCTT>5899
1195>~~~~~~~~~~~~~~~~~~~~~~~~~~~~~~~~~~~~~~~~~~~~~~~~~~~~~~~~~~~~~~~~~~~~~~~~~~~~~~~~~~~~~~~~~~~~~~~~~~~~>1195

     *         *         *         *         *         *         *         *         *         *
5900>TTCTGGATTCATCGACTGTGGCCGGCTGGGTGTGGCGGACCGCTATCAGGACATAGCGTTGGCTACCCGTGATATTGCTGAAGAGCTTGGCGGCGAATGG>5999
1195>~~~~~~~~~~~~~~~~~~~~~~~~~~~~~~~~~~~~~~~~~~~~~~~~~~~~~~~~~~~~~~~~~~~~~~~~~~~~~~~~~~~~~~~~~~~~~~~~~~~~>1195

     *         *         *         *         *         *         *         *         *         *
6000>GCTGACCGCTTCCTCGTGCTTTACGGTATCGCCGCTCCCGATTCGCAGCGCATCGCCTTCTATCGCCTTCTTGACGAGTTCTTCTGAGCGGGACTCTGGG>6099
1195>~~~~~~~~~~~~~~~~~~~~~~~~~~~~~~~~~~~~~~~~~~~~~~~~~~~~~~~~~~~~~~~~~~~~~~~~~~~~~~~~~~~~~~~~~~~~~~~~~~~~>1195

     *         *         *         *         *         *         *         *         *         *
6100>GTTCGAAATGACCGACCAAGCGACGCCCAACCTGCCATCACGAGATTTCGATTCCACCGCCGCCTTCTATGAAAGGTTGGGCTTCGGAATCGTTTTCCGG>6199
1195>~~~~~~~~~~~~~~~~~~~~~~~~~~~~~~~~~~~~~~~~~~~~~~~~~~~~~~~~~~~~~~~~~~~~~~~~~~~~~~~~~~~~~~~~~~~~~~~~~~~~>1195

     *         *         *         *         *         *         *         *         *         *
6200>GACGCCGGCTGGATGATCCTCCAGCGCGGGGATCTCATGCTGGAGTTCTTCGCCCACCCTAGGGGGAGGCTAACTGAAACACGGAAGGAGACAATACCGG>6299
1195>~~~~~~~~~~~~~~~~~~~~~~~~~~~~~~~~~~~~~~~~~~~~~~~~~~~~~~~~~~~~~~~~~~~~~~~~~~~~~~~~~~~~~~~~~~~~~~~~~~~~>1195
```

```
     *         *         *         *         *         *         *         *         *         *
6300>AAGGAACCCGCGCTATGACGGCAATAAAAAGACAGAATAAAACGCACGGTGTTGGGTCGTTTGTTCATAAACGCGGGGTTCGGTCCCAGGGCTGGCACTC>6399
1195>~~~~~~~~~~~~~~~~~~~~~~~~~~~~~~~~~~~~~~~~~~~~~~~~~~~~~~~~~~~~~~~~~~~~~~~~~~~~~~~~~~~~~~~~~~~~~~~~~~~~>1195

     *         *         *         *         *         *         *         *         *         *
6400>TGTCGATACCCCACCGAGACCCCATTGGGGCCAATACGCCCGCGTTTCTTCCTTTTCCCCACCCCACCCCCCAAGTTCGGGTGAAGGCCCAGGGCTCGCA>6499
1195>~~~~~~~~~~~~~~~~~~~~~~~~~~~~~~~~~~~~~~~~~~~~~~~~~~~~~~~~~~~~~~~~~~~~~~~~~~~~~~~~~~~~~~~~~~~~~~~~~~~~>1195

     *         *         *         *         *         *         *         *         *         *
6500>GCCAACGTCGGGGCGGCAGGCCCTGCCATAGCCTCAGGTTACTCATATATACTTTAGATTGATTTAAAACTTCATTTTTAATTTAAAAGGATCTAGGTGA>6599
1195>~~~~~~~~~~~~~~~~~~~~~~~~~~~~~~~~~~~~~~~~~~~~~~~~~~~~~~~~~~~~~~~~~~~~~~~~~~~~~~~~~~~~~~~~~~~~~~~~~~~~>1195

     *         *         *         *         *         *         *         *         *         *
6600>AGATCCTTTTTGATAATCTCATGACCAAAATCCCTTAACGTGAGTTTTCGTTCCACTGAGCGTCAGACCCCGTAGAAAAGATCAAAGGATCTTCTTGAGA>6699
1195>~~~~~~~~~~~~~~~~~~~~~~~~~~~~~~~~~~~~~~~~~~~~~~~~~~~~~~~~~~~~~~~~~~~~~~~~~~~~~~~~~~~~~~~~~~~~~~~~~~~~>1195

     *         *         *         *         *         *         *         *         *         *
6700>TCCTTTTTTTCTGCGCGTAATCTGCTGCTTGCAAACAAAAAAACCACCGCTACCAGCGGTGGTTTGTTTGCCGGATCAAGAGCTACCAACTCTTTTTCCG>6799
1195>~~~~~~~~~~~~~~~~~~~~~~~~~~~~~~~~~~~~~~~~~~~~~~~~~~~~~~~~~~~~~~~~~~~~~~~~~~~~~~~~~~~~~~~~~~~~~~~~~~~~>1195

     *         *         *         *         *         *         *         *         *         *
6800>AAGGTAACTGGCTTCAGCAGAGCGCAGATACCAAATACTGTCCTTCTAGTGTAGCCGTAGTTAGGCCACCACTTCAAGAACTCTGTAGCACCGCCTACAT>6899
1195>~~~~~~~~~~~~~~~~~~~~~~~~~~~~~~~~~~~~~~~~~~~~~~~~~~~~~~~~~~~~~~~~~~~~~~~~~~~~~~~~~~~~~~~~~~~~~~~~~~~~>1195

     *         *         *         *         *         *         *         *         *         *
6900>ACCTCGCTCTGCTAATCCTGTTACCAGTGGCTGCTGCCAGTGGCGATAAGTCGTGTCTTACCGGGTTGGACTCAAGACGATAGTTACCGGATAAGGCGCA>6999
1195>~~~~~~~~~~~~~~~~~~~~~~~~~~~~~~~~~~~~~~~~~~~~~~~~~~~~~~~~~~~~~~~~~~~~~~~~~~~~~~~~~~~~~~~~~~~~~~~~~~~~>1195

     *         *         *         *         *         *         *         *         *         *
7000>GCGGTCGGGCTGAACGGGGGGTTCGTGCACACAGCCCAGCTTGGAGCGAACGACCTACACCGAACTGAGATACCTACAGCGTGAGCTATGAGAAAGCGCC>7099
1195>~~~~~~~~~~~~~~~~~~~~~~~~~~~~~~~~~~~~~~~~~~~~~~~~~~~~~~~~~~~~~~~~~~~~~~~~~~~~~~~~~~~~~~~~~~~~~~~~~~~~>1195
```

```
      *         *         *         *         *         *         *         *         *         *
7100>ACGCTTCCCGAAGGGAGAAAGGCGGACAGGTATCCGGTAAGCGGCAGGGTCGGAACAGGAGAGCGCACGAGGGAGCTTCCAGGGGGAAACGCCTGGTATC>7199
1195>~~~~~~~~~~~~~~~~~~~~~~~~~~~~~~~~~~~~~~~~~~~~~~~~~~~~~~~~~~~~~~~~~~~~~~~~~~~~~~~~~~~~~~~~~~~~~~~~~~~>1195

      *         *         *         *         *         *         *         *         *         *
7200>TTTATAGTCCTGTCGGGTTTCGCCACCTCTGACTTGAGCGTCGATTTTTGTGATGCTCGTCAGGGGGGCGGAGCCTATGGAAAAACGCCAGCAACGCGGC>7299
1195>~~~~~~~~~~~~~~~~~~~~~~~~~~~~~~~~~~~~~~~~~~~~~~~~~~~~~~~~~~~~~~~~~~~~~~~~~~~~~~~~~~~~~~~~~~~~~~~~~~~>1195

      *         *         *         *         *         *         *         *         *         *
7300>CTTTTTACGGTTCCTGGCCTTTTGCTGGCCTTTTGCTCACATGTTCTTTCCTGCGTTATCCCCTGATTCTGTGGATAACCGTATTACCGCCATGCAT>7396
1195>~~~~~~~~~~~~~~~~~~~~~~~~~~~~~~~~~~~~~~~~~~~~~~~~~~~~~~~~~~~~~~~~~~~~~~~~~~~~~~~~~~~~~~~~~~~~~~~~>1195
```
